# Fortitude and resilience in service of the population: a case study of dental professionals striving for health in Sierra Leone

**DOI:** 10.1038/s41405-019-0011-2

**Published:** 2019-05-13

**Authors:** Swapnil G. Ghotane, Stephen J. Challacombe, Jennifer E. Gallagher

**Affiliations:** 1 Faculty of Dentistry, Oral and Craniofacial Sciences, King’s College London, Centre for Host Microbiome Interactions, Denmark Hill Campus, Bessemer Road, London, SE5 9RS UK; 2 Faculty of Dentistry, Oral & Craniofacial Sciences, King’s College London, Centre for Host Microbiome Interactions, Floor 22, Guys Tower, Guys Hospital, London, SE1 9RT UK

**Keywords:** Health care, Dentistry

## Abstract

**Objective:**

Sierra Leone (SL), with a population of over 7 million people, has a critical health workforce shortage. This research explores the views of key players on population oral health needs and demands, the challenges of oral and dental care delivery, and professional careers in dentistry, in order to inform future capacity building.

**Materials and methods:**

Semi-structured interviews were conducted with a purposive sample of key players in dentistry and healthcare, both in-country and externally. An interpretive phenomenological approach was used in exploring views of key-players on the oral needs and demands of population, challenges in the delivery of oral and dental care, professional careers of dental professionals in SL, and future workforce capacity building based on a topic guide drawn from the available literature. Interviews were audio-recorded, transcribed verbatim, anonymised and analysed using QSR NVivo 10 for data management and reported in accordance to the consolidated criteria for reporting qualitative research.

**Results:**

Twenty-one informants, of whom 18 were male, 17 were in-country and 16 were dental professionals, participated in the research. Dental professionals reported clear consensus on a considerable level of unmet oral health needs, most notably dental caries and periodontal disease, together with life threatening oral conditions such as osteomyelitis, Ludwig’s Angina and Burkitt’s Lymphoma. Challenges associated with the delivery of dental care revolved around five themes: patients’ predisposition for traditional remedies and urgent care; practical hindrances to the delivery of care; professional isolation and weak governance; and place with pressing local crises and lack of political will. An emerging typology of dental professionals included: demonstrating loyalty to their nation and family; exhibiting resilience in challenging circumstances; embracing opportunity most notably amongst expatriates; and striving to serve the needs of the population. There was support for innovative future capacity building developments.

**Conclusion:**

This paper provides important insights to the delivery of dental care in a low-income country with significant oral health needs and multiple challenges in the delivery of dental care, whilst also providing a vision for developing, building and retaining future human resources for oral health.

## Introduction

Sierra Leone (SL), in West Africa, with a young and rapidly growing population of over seven million people, is currently at bottom of the Human Development Index, recovering from the effects of civil war (1991–2002),^1,2^ a major outbreak of Ebola virus disease (EVD) (2013–15),^3^ and more recent natural disasters (2017);^4–6^ all of which resulted in heavy loss of life and highlighted its fragile health system.^7–10^

The United Kingdom (UK) has supported Sierra Leone on its journey from independence in 1961 through economic challenges,^11,12^ and the above crises,^13,14^ including the provision of international aid.^15^ King’s College London, a leading centre for global health, with a particular focus on sub-Saharan Africa, and King’s Health Partners (KHP), an academic health science centre in south east London, have played an important role in Sierra Leone over the past five years. King’s Sierra Leone Partnership (KSLP) within King’s Institute for Global Health,^16–18^ established in 2013, is defined by the philosophy of partnership working involving research informed action. Whilst King’s staff provide support from London, there is also a team of King’s personnel permanently based in Freetown to assist with a range of projects. One of the collaborative work streams relates to dentistry and oral health,^19^ following a request from Sierra Leone partners for assistance in tacking their ‘silent epidemic’.^16^

Non-communicable diseases (NCDs), which include oral conditions (most notably dental caries and periodontal diseases), present a challenge to health services and health policies around the world,^20,21^ in relation to their prevalence,^22^ and impact on population health and well-being.^23–26^ Oral conditions are amongst the most highly prevalent diseases globally with ‘untreated caries in permanent teeth’ reportedly seen in almost 35% of world’s population across all ages.^22^ The WHO has appealed to all dental organisations globally to work collectively for raising awareness against non-communicable oral diseases to support the ‘Global Action Plan for the Prevention and Control of NCDs’,^27^ and particularly in Africa.^28^ Disease control requires upstream health promotion and downstream management of disease, both of which require health personnel.^29^

Human resources for health (HRH) have been identified as one of the most vital part of the health system.^30–35^ In 2015, the numbers of physicians, nursing/midwifery and dentistry personnel reported per 10,000 population for high income countries (HICs) was ~28.7, 88.2 and 6.5 respectively; however, the numbers are significantly lower for low income countries (LICs) with figures of 7.9, 18.0 and 1.2 respectively.^36^ The severe dearth of HRH in the LICs is evident; however, the situation for human resources for oral health is even bleaker.^37^ Globally, the ratio of dentists to population in HICs is almost 1:2000; whereas in Africa it is about 1:150,000.^28,36^

The United Nation’s vision for 2030 is to achieve sustainable development goals (SDGs) through universal health coverage for all.^37–39^ This requires human resources for oral health,^39,40^ and the determination to revive the health system especially for resource restrained LICs.^41^ Workforce considerations must take account of population needs, together with a range of other factors in an integrated as well as a flexible approach.^42,43^ However, a critical barrier especially for LICs, has been the dearth of evidence or an understanding of local issues.^43^ Future planning needs to build on the realty that exists and learn from those who deliver care on the ground.

Within Sierra Leone, although the high need for dental care expansion was reported back in 1961,^44^ there is a dearth of recent epidemiological data on oral needs, even though unpublished and historical data suggest significant levels of unmet need.^45–48^ Dental services are very limited and fluctuate with up to 14 whole-time equivalent dentists, the majority of whom work in the private sector. Just three dentists currently work in the public sector,^16^ and only one public sector hospital has a fully staffed dental clinic (including four dental therapists) and is in the capital. SL, additionally, receives occasional visits from international dentists on short-term projects to provide pain-relief and deliver oral health promotion activities, the latter being funded externally.^49^

SL has never had a formal dental training centre, albeit there was a short period of dental therapy training prior to the civil war. They have, therefore, relied on a small number of dental personnel, mainly dentists, trained abroad.^16^ This shortage of skilled labour parallels that of sub-Saharan Africa as whole, where there is limited education and training of health personnel and excessive migration to high income countries (HICs).^50^ This is exemplified by the fact that whilst Africa bears almost a quarter of the global burden of disease it has less than four percent of the health workforce available.^51,52^ The importance of using community-based and mid-level providers to expand capacity in health care within Africa,^53,54^ is increasingly recognised and dental care is no exception.^54,55^

Whilst there has been some research on migration, no study, to the knowledge of the authors, has examined the views of dental professionals who work in low-income settings as part of a wider project to inform the development of a sustainable human resource for oral health. Prior to considering the establishment of a dental training centre to support workforce expansion, it is important to explore the motivation and careers of existing personnel in dentistry, their recruitment and retention in the country, their perception of the oral needs and demands of the population and the challenges of delivery care, together with their vision of how the needs of the country may be met. Whilst such research focuses on dental personnel, the views of other key players within the health and education sector are also important.

The aim of this study was, therefore, to explore current needs, demands and challenges of delivering oral care in SL together with dental professionals’ careers in dentistry, their recruitment to, and retention in, a low-income country addressing the following research questions:

1. What are the views of key players on needs, demands and challenges of oral care delivery in SL?
2. What are the views and experiences of dental professionals on their professional careers in SL?
3. What are the implications for planning and developing a sustainable model of educating, training and sustaining human resources for oral health in future?

## Materials and methods

Semi structured interviews were employed to explore the views of dental professionals and health care leaders in addressing the above questions and exploring social processes and contexts that shape healthcare^56–59^ in line with an interpretive phenomenological approach. Ethical approval was gained from King’s College London (KCL) Research Ethics Committee (LRS-14/15-17320) with minor amendments (RESCMR-15/16-1732; RESCM-17/18-1732) to gain time extension and invite additional participants related to the field of dentistry who came to the research team’s notice as the study progressed. Ethical approval from ‘Sierra Leone Ethics and Scientific Review Committee’ was sought and obtained in parallel, as the study included participants’ from both Sierra Leone and the United Kingdom [UK].

The study was based on, and conducted in accordance with Tong et al. consolidated criteria for reporting qualitative research.^60^ A topic guide, informed by the literature,^61–64^ addressed the research questions outlined above was divided into four sections as follows:

- oral health needs and demands
- the challenges of delivering care;
- the professional careers of current dental workforce including career interests and goals, and
- their future vision for Sierra Leone.

### Sample selection

**Fig. 1:**
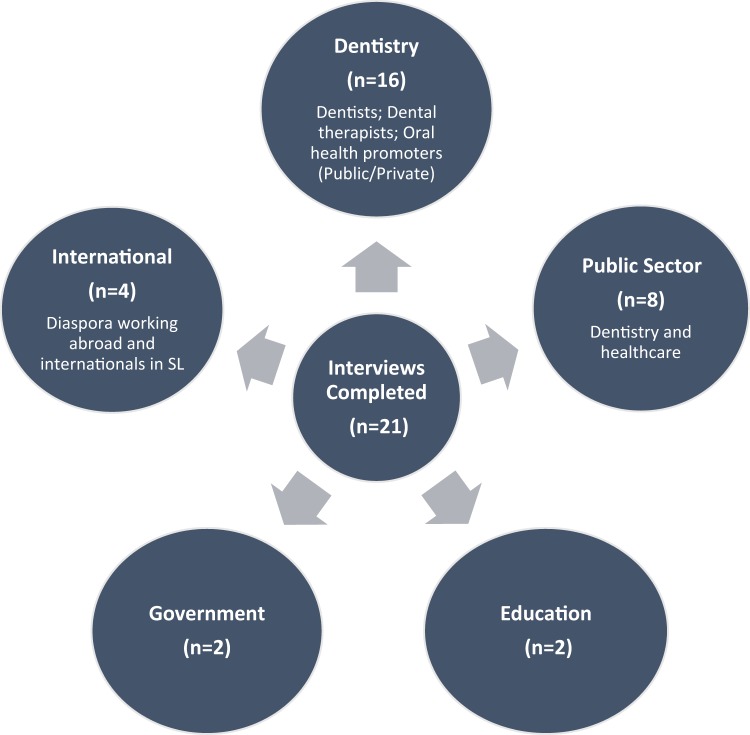
Interviewees from different sectors in SL and the diaspora. Note: interviewees represented more than one category

A purposive sample of people with a knowledge of dentistry,^65,66^ was invited to participate, based upon their role, experience and expertise in relation to SL. This included key players whose involvement (either directly or indirectly) was within healthcare and related policy. King’s Centre for Global Health, with a permanent base in the capital city Freetown, facilitated in identifying and making strategically relevant contacts.^1^ The sample included participants from current dental workforce in SL, ministries of ‘health and sanitation’ and ‘education’, the public healthcare university, public sector hospital, international organisations supporting oral health, and the SL diaspora (Fig. 1), with some participants holding overlapping roles. Interviews were conducted between October 2015 and December 2017.

### Sample recruitment and interview process

Participants were contacted by phone or email, the former being the prime mode utilised in-country. At first contact, invitees were provided with an information sheet and consent form, having at least 24 h to decide on their participation. Follow-up contact (by phone or email) explored their willingness to participate in the research. For those interested and willing to be interviewed, a convenient date, location and mode (face-to-face, by Skype or phone) were agreed in line with their preference. Out of the 21 interviews, 19 were conducted by SG (doctoral student) and remaining by JEG. SG had no prior collaboration with any study participants. The duration of the interviews ranged from 30 to 90 mins and written consent was obtained by the researcher (SG or JEG) before commencement of the interview. Consent was rechecked prior to commencing the interview. The researchers informed the participants and took notes during the interview. Interviews were audio recorded and transcribed verbatim. Participants were informed that they could request a copy of their transcript to check for accuracy and suggest any modifications.

### Data analysis

Data were analysed using an interpretive phenomenological approach (IPA),^67^ which has been used and tested in healthcare research.^68–71^ IPA helps the investigator to explore personal meaning and lived experience of participants, and justification that participants make for experiences that they have been through.^72,73^ In this study, it was crucial to explore views of participants within context of national crises such as civil war and Ebola virus disease and their personal experiences in that context. In addition, IPA was helpful as it provided a ‘critical realist approach’,^68^ on how participants perceived dentistry as a career within SL context. IPA is a cyclical process with four iterative stages: first, familiarisation with the raw data, followed by identifying preliminary themes (second stage) which are then grouped together as clusters in the third stage; and, finally, creating a summary table to conclude the findings or theory emerging.^74^ This study followed the above approach in turn commencing with reading and re-reading the transcripts, checking and anonymising the data (SGG and JEG.). Following data familiarisation, a coding thematic index consisting of main- and sub-themes was developed based on the emerging text and informed by relevant literature.^75–80^ Themes were further crosschecked by the research team, and differences resolved through discussion. The data were systemically analysed and ordered using Excel and QSR NVivo 10 software. The thematic index informed an emerging theory on building and sustaining a suitable dental workforce in SL along with a typology of dental professionals.

## Results

### Interviewees’ characteristics

A total of 35 participants were contacted from October 2015 to December 2017, out of which 23 consented to participate, 21 of whom were available for interview. The majority were male (86%; *n* = 18), in-country (81%; *n* = 17) and from a dental background (76%, *n* = 16). A higher proportion of non-responders (*n* = 12) were female (75%, *n* = 9); one participant withdrew due to illness whilst another declined without providing a reason. Amongst those with formal training in dentistry, 63% were dentists (*n* = 13) and 14% dental nurse/therapists (*n* = 3). A minority of other healthcare workers were involved in the field of dentistry, mainly in a complimentary role (10%; *n* = 2). All participating dental professionals received their qualification abroad across a wide range of countries from Europe (UK, Russia, Germany, Hungary) to Syria, India, New Zealand and Cuba. The remaining participants were from the Ministry of Health and Sanitation, and the Sierra Leonean diaspora (14%, *n* = 3).

### Dentistry: as a profession and a work opportunity

Participants reported that the delivery of oral care has been limited as the number of dentists over last five decades has ranged between 5 and 14 dentists, with almost all based in Freetown,^2,44,81,82^ with some holding other responsibilities. They expressed strong concern over much of the population having unmet oral health needs, whilst capacity in oral care delivery was limited; this reportedly acted as a ‘motivator’ for participants to undertake dentistry as a profession (Table 1). Sierra Leoneans expressed a love for their nation and those working in the country reported that their passion to ‘serve their people’ never declined. This immense feeling of pride associated with serving the country was evident through participants’ empathy for fellow citizens and a strong desire to relieve them of ‘pain’ as exemplified by the following quotation from a dentist working in-country:

“…my main motivation of going to dental school was that I would be able to be back home someday to help the people there, because there’s a very big need… I saw women and children, they’d come over to the clinics overnight. They sleep there because they want to be among the few people that will be selected to see the dentist the next morning”…(D_9)

**Table 1:** Striving to care: journey of oral health care in Sierra Leone

Phase: time-period description	Main themes	Sustained challenges	Quote
Building: 1961–1989	Desire to serve people in need Support to train in dentistry Integration into government service Modest dental care and dental professional body	Migration Corruption Limited resources	*“Well the motivation was you know we have a lot of people in our country suffering from this teeth problem… (D_1)”*. *“I came straight from school … it was an advert in (paper)…in SL (for dental nurse). I applied and was told to go to Ministry of Education…I got a scholarship” (DT_1)*. *“When I came back the country needed dentists by then. So to process our papers and then enter the Government service it was not difficult at all. In less than six months that was settled… (D_8)”*. *“(SL) had full available facilities with quite a number of lab technicians, they had quite a number of dentists and they also had what they called dental nurses…(NG_1)”*
Following independence & prior to civil war			
Brutal: 1990–2001	Personal crisis Surviving strategies Depletion of dental workforce Cessation of dental care	Migration Corruption Limited resources	*“Well that period was, I think bad in itself because we were working on the gun points more or less and after a while I just left, I just closed down and I left after about 6 months, I gave it up because it was useless…(D_7)”* *“So if I don’t come back who will come and do the dirty job for us?…I have internal satisfaction… you know, from 1994 to now the number of lives I have saved…(G_1)”*
Civil war			
Bereft: 2002–2013	Economic challenges Political apathy towards dentistry Opportunity for work International aid for dental care	Migration Corruption Limited resources	*“..the government they don’t want to spend on health. …it is not their priority, they (government) don’t give two hoots… doesn’t matter how many people die… they don’t pay attention to dentistry (D_3)”*
Recovery period after civil war			
Bleak: 2014–2015	Coping strategies Fear Atmosphere Economic challenges Intermittent dental care	Migration Corruption Limited resources	*“It was a challenging experience, but more than challenging, I would say it was a learning experience for me, because I have never worked in such kind of environment to be honest… (D_2)”*
Ebola period			
Re-Building: 2015–current	Economic challenges Awareness for protecting oneself Shortage of dental resources Decrease in International aid	Migration Corruption Limited resources	*“…that was a consensus that everybody had to put arms to fight Ebola, it is, it has drained the economy of SL really because for about two years things were not working… but one thing I am sure it has left with us is that we have become aware that we should protect ourselves and I think that is the key element I would leave with everybody … that if you want to be free from this kind of communicable diseases, protection is best solution…(D_6)* *“… things were really difficult after the Ebola. But now we just wait for people who are assisting the government. Because lately we’ve seen the government strain. And after the Ebola now we are just trying to recover and that is why things are very, very difficult…(DT_3)* *“Because before this time, before the Ebola, there was no triage system. But after the Ebola now, there is the triage system wherein the patient goes to the triage…(DA_3)”*
Post-Ebola period			

Not all participants delivering dental care in-country were nationals. Some expatriate dental professionals reported SL as providing a unique opportunity (Table 1) to help people who have limited access to dental care and are in dire straits due to dental disease, including saving lives as shown below:

“In fact, I was really keen to work in a country I’ve never been (to), and it was really a good opportunity for me. So, I came over here, and yes, like I said, it has been a really wonderful experience since I have worked here”…(D_2)

“There is no help …my mission is to save life…when I go out there (outside the capital of SL) I try to save as many lives as I can…but I also sort of get angry about the fact that people die because of a simple decayed tooth…I do not accept that children are dying because of a single decayed tooth. I have seen people dying of dental disease and I have captured (such cases on film) …some of them we could save and some we couldn’t”… (NG_3)

In addition, some expatriate dental professionals reported the ease with which immigrants were able to practice dentistry in Sierra Leone compared with having experienced stringent regulations elsewhere as suggested by the following quotation:

“Europe and even Arab countries they don’t give you a visa…only after I complete course they give you a Visa…only Africans they give you Visa…when nobody give me a place or something, they (SL) give me here a place, I can make money here, I live with my family, my life, I lived here with dignity when nobody came to me”…(D_5)

### Support to train and retain dental professionals in SL

As historically, Sierra Leone did not train dentists, participants described how, instead, the government sponsored local candidates to purse their professional education and training in dentistry outside of the country. Out of the 10 SL born dental professionals (dentists and dental nurses/therapists) participating in this study, seven had received government funding to train abroad; and, thus, dentistry was reported as a career opportunity:

“I think it was the early eighties; it was not the kind of profession that I was thinking of… Fortunately there was an advertisement to study dentistry in (place). So, I actually applied…but I wanted (…) to study medicine, but then (…) the panel there, some of them were saying (…) we don’t have dentists in Sierra Leone, don’t you think it would be nice for you to go and study dentistry. So, I accepted the offer. That was how I actually entered - came - into dentistry”…(D_8)

Even though the number of dental professionals was low, the Government was reported as actively supporting the process of building capacity for oral health care in the pre-civil war era (Table 1). The Government was not only funding people to train abroad but also incorporating these professionals into public service after their return to SL ensuring that the population had access, albeit limited, to dental care. Participants reported that there was at least one dentist in each of the four regions of Sierra Leone at that time:

“…when I went to Sierra Leone there was a very organised dental service which consisted of the main headquarters in Freetown and clinics in the eastern and western part for paediatric dentistry and services in various parts of the country”…(NG_1)

In addition, participants reported effective governance which ensured all dental professionals working in SL were qualified and delivering quality dental care prior to civil war, with an active ‘Sierra Leone Medical and Dental Association’ (Table 1).

“In the initial stages, we used to have an association, dental and medical association …especially the dental society which is a special body only for dentists…it was also a disciplinary body, it was (….) to check on your qualifications so I remember when I came (to SL) I had to work under a senior colleague for (…) almost two years” …(D_3)

One important issue highlighted by the participants was related to the availability of resources (both materials and personnel) (Table 1). Mixed views were reported as some participants acknowledged the availability of dental resources while some disagreed. In addition, managing with available resources was also a challenge with some dentists arranging for dental resources privately even in the public sector:

“Well the difference basically is there was nothing to work with in terms of equipment and supplies, even in Freetown there was hardly anything you could work with… luckily for me I had brought everything with me and I equipped the whole surgery in the government service where I was working” …(D_7)

### Survival and endurance

Participants expressed their sorrow over the decade long civil war (1991–2001) which had devastating effects on the whole country, halting government capacity building for oral healthcare (Table 1). Dental personnel who endured the civil war era highlighted the loss of capacity during that phase and a range of challenges in delivering oral healthcare. The atmosphere of constant fear during the civil war prompted dental personnel to leave; however, some participants reported returning because of family responsibilities, as follows:

“At the civil war we had to stop it, we had to shut down each centre. I left to work in [country]…Yes, I was there for a year and then I came back (to SL), started all over again…I’m the eldest in my family so I have dad and mum getting old…how could I leave them, and I have younger siblings…I have to stay…I have a family I need to support”… (DT_1)

One of the crucial factors reported by participants during, and after, the civil war period was the widespread corruption, together with their anguish on the neglect of oral health care and dental personnel in SL by the political fraternity. Consequently, participants highlighted the issue of reduced funding to train the dental workforce abroad.

“…the main thing was significant deterioration in the economy and that was due to political mismanagement…there was massive corruption and you know funding that should have gone towards services, whether it’s the education, health, transportation sector was diverted to people’s own personal use” …(NG_1)

“It’s not like before when they (SL government) gave a lot of scholarships. Well it changed during the war”…(D_1)

Following the civil war, participants reported a lack of improvement in the organisation of oral health care (Table 1). Apathy and uncertainty continued over the period, which resulted in the further stagnation of dentistry. In addition, participants reported feeling dejected as the professional body ensuring the quality of dental care became non–existent.

“We had a Dental Society before, sorry, Dental Medical Association for a while, that has gone to the dogs for many years…I suppose because of loss of interest, people went away…they left, they retire and some of the retirements should not stop you from participating in the professional association”…(D_4)

The Ebola outbreak in 2014 further compounded issues for SL. Participants reported the sudden onset of Ebola crisis as a challenging experience because there was a constant atmosphere of fear and extra measures for infection control and personal protection were needed to deliver dental care safely. There was a temporary closure of health and dental services during this period (Table 1); however, some dental personnel were able to practice after receiving training in personal protective measures against Ebola infection as reported by one dentist below:

“Well for example I shut down … until things got so bad that I told my wife I cannot sit here, I have to go and so we came in the midst of it (Ebola) and I went to the PPE course in which they were training doctors…how to handle Ebola” …(D_3).

### Oral health needs and demands in SL perceived by participants

Twelve dental participants had considerable experience (more than 15 years) of working in Sierra Leone during which time they mainly treated the effects of dental caries and periodontal disease. Respondents were concerned by the availability of sugar and its implications for oral health as follows:

“Cheap unhealthy sugary diet which is so available…more than fish or meat…it is so readily available…especially in the districts I would say…for e.g. if you are travelling within Freetown you can see people on the roads pushing packets of biscuits to sell you…they are very cheap” …(NG_3)

“Well the common problems yeah, mostly it’s gum problem - that is, gingivitis. We have a lot of dental calculus and we have a lot of this dental periodontitis, […] mobile teeth and then more of dental caries” … (D_1)

In addition, all dental professionals expressed their concern regarding poor oral health among the population and their preference for extractions to get rid of the pain. Furthermore, life threatening conditions such osteomyelitis, Ludwig’s Angina and Burkitt’s lymphoma were not uncommon in SL, despite being rare, or non-existent, elsewhere.

“…oral health is very poor in this nation, the people don’t care about their dentition, they don’t care what, so like they go in more for extractions” … (DT_2)

“…there is no district where you won’t see a Burkitt’s lymphoma or Ludwig’s Angina or osteomyelitis” … (NG_3)

“Like Ludwig’s I said earlier on, Burkitt’s lymphoma [PH], osteomyelitis, those ones I can spot for myself and know that this is this. So, I will be referring based on what I’ve noticed. But the most common ones as of now are Ludwig’s and osteomyelitis” …(DA_1)

One participant outlined how the oral needs of people were linked to cultural traditions and socio-economic status as follows:

“…there was a lot of prosthetic work as well which obviously following from the extractions because if people have a lot of teeth out they need to function so they need prosthetic work so there was a lot of prosthetic work and a lot of crown and bridge work as well because also part of the culture at that time as part of the status symbol people liked gold in their teeth so if they needed a crown they wanted a gold crown and they wanted it to show that there was a lot of crown and bridge work”…(NG_1)

### Challenges of practising dentistry: striving for health

**Fig. 2:**
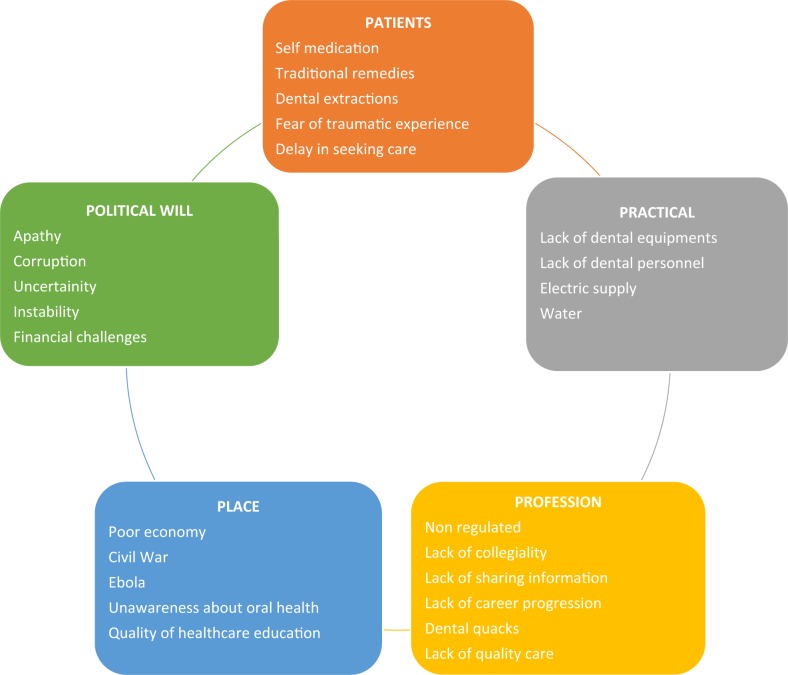
Challenges in oral health care in Sierra Leone

Challenges reported by the participants in delivering care, revolved around five major themes relating to patients, practice, profession, place and political will which demonstrated the overarching theme of striving for health (Fig. 2).

#### Patients’ predisposition for traditional remedies and urgent care

Participants reported that preferences of patients (people in SL) were one of the main challenges for dental professionals. The tendency to self-manage disease, use traditional medicine and seek care from traditional healers, complicated the oral health issues among the people, fuelled by beliefs that oral pain and swelling are the result of witchcraft or spells.

“People will put some medication, sometimes like paracetamol, sometimes people will put camphor, they put in there, in the tooth just for them to cease the pain. If the pain can cease, then they will not go to the clinic”… (D_9)

“They believe in witch doctor more than they believe in therapeutic care, somebody who is actually more qualified, and the reason for that is the witchdoctor is cheap and, also, they have this aura of healing people but actually they kill people” …(NG_3)

“They would just use their local…what you say (witchdoctor), in fact when you see (an) ameloblastoma they will tell you that it’s cultural, they have this medicine men who would put something on, it’s a spell or something, you have to really convince them to come (to the dentist), yes they all have their issues, yeah terrible issues and things like that. If you tell them they would say no…it is by witch curse, you know their native beliefs” …(DT_1)

“…also, these people in the villages or in some big towns in the provinces, what they do is they go to the traditional healers or sometimes they treat themselves with native medicines… they boil it, put it in their mouth and that’s why some of them after a few times start having [a] problem” …(D_1)

Interviewees felt that patient preferences for self-medication and traditional remedies could be mainly linked to the very limited access to dental care (available only in Freetown) which was further accentuated by the poor economy. Also, some participants highlighted the element of fear in accessing dental care due to previous traumatic experience as children, as follows:

“Well, first of all because we have limited number of doctors who are dentists in this country, we have many challenges, especially in the provinces. Most of the provinces now, most of the big towns in the provinces, or even the villages there is no dentist. There is no dental therapy, there is nobody to give them, I mean some kind of dental cure”… (D_1)

“Exactly, and of course, many have […] economical problems. They want the care. They truly want the dental care, they want to keep the oral hygiene, but because of the economical problems, since this is one of the poorest countries, they are like … they hesitate, and they are not prepared to come to the dental facility”… (D_2)

“Well there are several reasons (…) some people who don’t have any trust in the health system. Sierra Leone doesn’t have any paediatric dentists; we don’t have anyone who practices paediatric dentistry or anyone who is specialised in that area of dentistry. So, people at a young age sometimes they’re having traumatic experience going to a dental clinic and then they lose confidence, and then we have the fear factor”…(D_9)

“Oh, I have problem, well basically people wait until things are really bad before they, come to see you…they prefer to get off with an extraction, never mind what happens to the dentition afterwards, than to have their teeth filled or crowned as the case might be”…(D_7)

#### Practical hindrances to the delivery of care

All participants expressed their difficulties in running a dental practice in Sierra Leone. One of the considerable barriers was severe lack of dental resources which compelled them to import all dental materials and equipment from outside of the country. Participants talked about how this not only affected their work abilities but also added to the financial pressure of practicing dentistry as outlined below:

“It’s very difficult for you to have dental materials in this country for you to buy. So sometimes you can only get them out of the country …send somebody to Guinea to buy them or maybe Nigeria… but in Sierra Leone you cannot get a dental material shop that’s the problem”…(D_1)

“…one of the biggest challenges we have is material to do the work, materials to do the work, because you may have the idea, you know …you have, there is a case in front of you, you know this is how I should go about it. But on the other hand you look, you don’t have this, you don’t have that, how do you go about taking care of that situation” …(D_8)

In addition, participants highlighted a lack of support staff in dentistry as one of the major barriers to the delivery of appropriate care:

“Also, we don’t have technician…and we don’t have technician here and we don’t have lab. We don’t have lots of things. Even dentists we don’t have dentists” … (D_5)

“We need dentists but in fact other saddest story that you won’t believe is that there is no dental technician. There was one who just got his retirement letter” … (DT_1)

“You need first, you need the personnel to upgrade oral health generally speaking there is little or none… you need quite a number, a good number of personnel in oral health management in the country” …(D_7)

Participants also reported the practical barrier of irregular power and water supplies, which can happen mid-treatments and considerably affect clinical practice as suggested by the following dentist:

“To do, now, when I have to do [a] restoration [dental filling] … [the] light comes and goes … I want this chair to work and it won’t” … (D_5)

#### Professional isolation and weak governance

One of the important challenges perceived by participants was current non-regulation of dentistry as a profession and they believe it has affected the quality of dental care.

“… I will say the population is not poor … but the population is poorly managed … and that bring us to the fact that laws are not binding people enough to know exactly what they should do, example there are a lot of people who are around with unsterile instruments and things like that and do extractions at random, infecting people with HIV and other transmissible diseases” …(D_6)

“No discipline, no discipline … there is no discipline … you don’t know who is who here, people come in like the other dentist and they say oh they know me, but I’ve never seen the light of day and they never introduce themselves … [no] they don’t do and I meet you […] who are you … oh I am the dentist yes … instead of me embracing him like a colleague [yeah]; that has gone…you understand” …(D_3)

In addition, some participants also reported their plight in not having continuous professional development (CPD) opportunities, which resulted in stagnation of their skills and thus affect the quality of care as suggested by the following dental therapist:

“… you remember from 2012 you never going to any ‘in service’ courses because (…) government are not putting in place those things. So, if we can have ‘in service’ courses or a refreshment training…that’s one thing I would suggest. Because you’ll be able to recall what sometimes you have forgotten. And during our stay when we are learning in school we see (that) when we come back the materials were not there. So, some of those procedures we won’t be doing again. So, we need to refresh ourselves…we can do a lot to improve the system. And we need to refresh our people’s memory again”…(DT_3)

#### Place with pressing local crises

Participants reported local issues which pose a challenge to dental professionals. First, the general lack of awareness in this low-income country of the importance of oral health and oral healthcare; therefore, effort being required to explain and educate the population as suggested below:

“You have to do a lot of motivation like you have to teach them to do regular cleaning and flossing and that sort of thing, you see, so oral health is not being taught, generally speaking”… (D_7)

“So when I was assisting (in clinic) it was like a pain in my head…because some (people) will come (to clinic), and in fact some (people) will (ask to)extract all the incisors…they don’t have the knowledge of it that they should be taking care of it (teeth), no oral health education has been given to them, they don’t have idea of it”…(DT_2)

Second, crises such as the civil war and Ebola has had a considerable effect on both patients and professionals and disrupted what service provision existed as described below.

“The civil war [yeah] that made me fear that we might…(die)…therefore I went back to (safe place), sat down there for some time and came back. My motive was that well my children could not stand seeing guns, and all those sort of things and my wife too was not very helpful…so I took them all to (place) and kept them there … for some time and then I came back” … (D_3)

#### Lack of political will

The level of political will associated with tackling dentistry was reported as a considerable barrier nationally. Participants suggested that this has compounded the dental care crisis, allied to corruption, uncertainty, instability and lack of financial support for this aspect of healthcare:

“Well, again, I think it depends on people who are in charge, they have to be pushed and there has to be the political will also, because in our part of the world, politics plays a big role, if you have somebody who could really push things through the political corridors, then they’d be able to listen, look here and say, yes, we need these” …(D_4)

“…if it’s out of our way to have let the medical and dental association be I mean they decide what is happening within and without any politics it would have been nice [yeah]…but when politics interfere, when the government chooses who it thinks is more or less politic uses that as the whole thing becomes more politicised rather than medicalised” … (D_3)

Healthcare in general, and oral healthcare in particular, were not considered a priority for the country; an issue, which was a source of frustration for some respondents:

“…so, it’s not only dentistry, it’s the whole general medical and health sciences … it is not their priority, they don’t give two hoots doesn’t matter how many people die [yeah] to them it’s just one, one, one of those thing of the day [yeah] and that is it … they don’t pay attention to dentistry”… (D_3)

### Future vision

Given the evidence of need amongst the population and challenges faced by dental professionals in delivering care, participants expressed a clear need for action that learns from the past and builds on the commitment of a few for the many as outlined below:

#### Building workforce capacity

Looking to the future, the need to develop a workforce was acknowledged by health professionals, academics and those in the ministry with a vision of developing dentists and dental therapists or equivalent mid-level providers, this not being mutually exclusive as demonstrated by the following quotes:

“My aim is to form [a] dental school…but we are not aiming at the moment for dentists…we can’t…we can only aim what we can achieve and that is (dental) therapists… (dental) therapists can take teeth out and give oral hygiene instructions…they can teach people about reasons of tooth decay and they can actually do a lot…therapists are amazing…they are very good people” …(NG_3)

“I think we need to bring in some people here, if you go to (place) there are so many specialists there. You can go to (place), you can bring in some people for a short time to help, I know a lot of colleagues, the guys in (place), I worked with, I trained with some of them, I worked with some of them, so I know them. Maybe you’ve got to bring people on short term to help, that’s the way I see until we start developing our own curriculum”…(D_4)

#### Infrastructure

In order to support capacity building, appropriate infrastructure was required:

“…improve even the current structures, infrastructure as you can see here, the dental unit is almost dilapidated. If you can compare the Obstetrician and Gynaecology, just close to us (dental) here, if you watch now the structure here with that over there, you see this one is just like a mission. Because people are not interested, and I can say, top that with that fear that even the government is not paying attention to it” …(DA_3)

#### Task shifting

Task shifting was considered by many respondents to be an important initiative for the future delivery of care:

“So, we have to do some task shifting. We have to provide some training locally. You know, capacity building here locally. Train, even CHOs [Community Health Officers – in dentistry]. We have these clinical officers who are trained from our schools here who can do in service training for them to build the capacity. It is more cost effective”…(G_1)

#### Scholarship

The value of scholarships to study for a primary or secondary qualification was considered important, either in-country or externally as suggested by the following quotations from different dental team members:

“[The] money’s not there so we’re actually hoping for scholarships to go and study out (of Sierra Leone) but if that is given definitely we can get involved and try to get ourselves equipped and from there we can start”…(DA_2)

“I want to go back, I want to study more…in dentistry…it will improve me…yes more skills” …(DT_2)

#### Remuneration

Remuneration was required to support any developments, notably for dental workers as suggested by a dental therapist:

“Somebody working in (place) would get good pay, good job, job satisfaction…do you understand, somebody working with those people (ministers), they have cars they have this that and we have nothing. They give them houses, they give them driver… they don’t give us anything, you see, and we are professional doing the hard work, you have to be encouraged. I think that’s the key, encouragement for the medical profession”…(DT_1)

### Emerging theory and typology of dental professionals

The findings from this innovative and important study provide important evidence of striving to address needs in a low-income country which has suffered catastrophic crises, where oral health care capacity has always been limited, whilst needs are significant and life threatening.

**Fig. 3:**
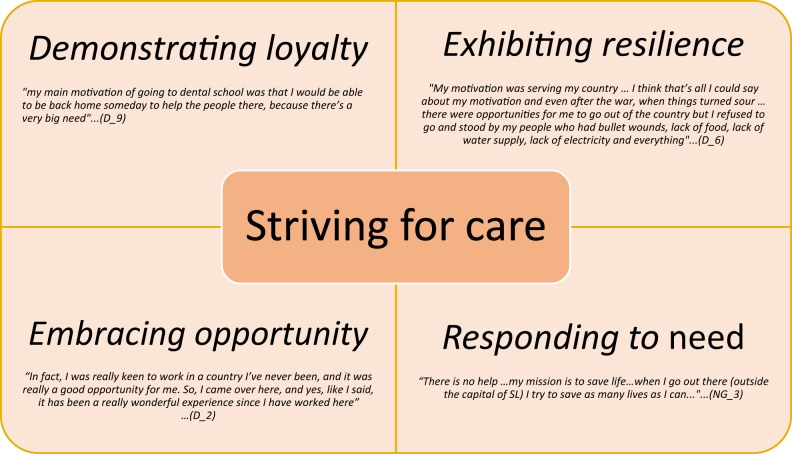
Typology of dental professionals working in Sierra Leone

Emerging typology suggests four types of dental professionals which are not mutually exclusive: demonstrating loyalty; exhibiting resilience; embracing opportunity and responding to need (Fig. 3). A common theme evident through this study was that of an immense loyalty of Sierra Leoneans towards their family and friends in country. Whilst the participants outlined the challenges of oral health care delivery, Sierra Leoneans valued serving fellow citizens, together with being able to carry their family responsibilities even during adversity, reflecting their loyalty and resilience in doing so through challenging circumstances.

Sierra Leoneans welcomed the opportunity for dental training and expatriates welcomed the professional opportunities provided to them personally and could in turn lead to demonstrating loyalty; they expressed a love for their nation and a powerful desire to help their fellow citizens. This suggests the importance of training a local workforce in responding to need, particularly in rural areas.

## Discussion

This is the first study, to take a qualitative approach exploring the context to inform future capacity building for dentistry in a low-income country in sub-Saharan Africa. The findings suggest considerable unmet routine oral health needs (dental caries and periodontal diseases), together with rare and life-threatening oral conditions such as osteomyelitis and Ludwig’s Angina, which result from untreated common diseases.^83–85^ Furthermore, Burkitt’s lymphoma, primarily seen in young children especially in Africa and commonly presenting as a tumour affecting the jaws,^86–88^ was also reportedly seen on a frequent basis. The study highlighted significant challenges within SL which mainly revolved around five major themes: patients’ predisposition for traditional remedies and urgent care; practical hindrances (irregular dental and infrastructure resources) to deliver care; professional isolation and governance issues of dental personnel; place with pressing local crises such as the civil war and the Ebola epidemic which had disastrous effects; and political will in tackling dental care issues. The findings suggest a typology for dental professionals which are not mutually exclusive: demonstrating loyalty; exhibiting resilience; embracing opportunity and responding to need which could be harnessed for future planning of services.^89^

### Push and pull factors for dentistry

The findings from this study highlight some of the ‘pull factors’ for entering in dentistry in SL as perceived by the respondents. Opportunities which presented, together with the desire of helping fellow nationals were reported as important factors for participants to choose dentistry as a career in SL. Moreover, participants either practising in SL or aspiring to return from abroad, conveyed ‘family responsibilities and influence of friends’ as major influences. Family/friends and altruism have been reported in various studies across the globe as one of the important influences for choosing dentistry as a career.^58,90**–**94^ Dentists are the lynchpin of the dental care system and therefore, consideration should be given for careful selection of a cohort of students to be trained externally who will return to provide care in their country.

In addition, participants reported that high levels of oral health needs and lack of human resources to address them provided a unique opportunity to work, not only to help people in need, but also working with limited professional competition. For some, it was the simplicity of regulations to enter and practice dentistry in SL that prompted their decision. However, participants also mentioned factors which act as deterrent (push factors) to pursue dentistry in SL. First, the lack of a conducive working environment and resources. Second, financial paucity due to widespread corruption and crises such as the civil war tends to push professionals outside of SL. These findings are in agreement with the issue of inadequate resources in oral health especially in low income countries documented in the recent WHO strategy for oral health in Africa.^28^

### Need for sustainable models of care

The study reported a will to examine new models of care to ensure that services are routinely available outside of the capital city and that care is relevant to the context. In this case, there is a strong argument for resurrecting the dental therapy training which had been planned some time ago. Since this research was conducted there is emerging evidence of an NGO seeking to deliver add on training for community health workers up-country to ensure that they have skills in dental care. All these approaches have much to offer and should be actively explored as evidence suggests using an integrated and flexible approach,^42^ considering the challenges of working in SL especially outside of the capital city.^95^

In addition, this study also highlights the underrepresentation of the female voice which has been a significant issue especially in low-income and post conflict countries.^96^ Future action is needed to promptly recognise gender barriers and encouraging female participation in developing best approaches for addressing needs of vulnerable people.^96^

In summary, the findings from this study are in line with those reported by Wurie et al. highlighting the demotivating factors for retaining health workers in SL.^75^ In addition, the findings from this study also resonates with the framework developed by Willis-Shattuck et al. on retaining health workers in developing countries adding the dimension of ‘influence of family and friends’ to their framework which highlighted financial incentives, career progression opportunities, working environment, availability of resources, management and personal recognition as core themes.^76^

### Strengths and limitations of study

The study provides evidence on the current and previous issues of oral health care delivery in SL. It encompassed key players from a range of sectors within SL and explored their views on oral health issues and capacity building in SL. This included a diverse sample from different important sectors across all four regions of SL and the diaspora. Although this study included most of the relevant dental professionals, one limitation is the lack of a population/patient perspective. The importance of involving the population in health services research and future planning is supported by the research team and this study is the first of several in country with normative and perceived needs to be explored in further research. The advantage of the qualitative method is the provision of exploration of the complex ideas and beliefs that key players may have on oral needs and sustainable workforce within the local context of SL.^26–28^ Moreover, qualitative approaches are responsive to local situations, conditions, and key players’ needs and will therefore, facilitate in exploring the phenomena based on the participants’ own understanding of them. Furthermore, an important dimension in qualitative data analysis is ‘reflexivity’ to maintain an ‘empathic neutrality’ in analysing data.^77^ The researchers in this study were trained dental professionals having experience of conducting qualitative research;^58,59,97,98^ however, they had no collaborative work in Sierra Leone prior to this study which helped to maintain a neutral point of view in analysing the data.

## Conclusion

This paper provides important insights to the delivery of dental care in a low-income country (LIC). It highlights significant oral health needs of SL and multiple challenges associated with patients’ predisposition, practical difficulties, professional isolation, place with pressing national crises such as civil war and Ebola, and lack of political will in delivery of dental care. In addition, the study reports Sierra Leoneans’ vision for building and retaining future human resources for oral health which could have implications for other post-conflict or LICs.
